# Extracellular Vesicles, A Possible Theranostic Platform Strategy for Hepatocellular Carcinoma—An Overview

**DOI:** 10.3390/cancers12020261

**Published:** 2020-01-21

**Authors:** Igea D’Agnano, Anna Concetta Berardi

**Affiliations:** 1 National Research Council (CNR), Institute for Biomedical Technologies (ITB), 20090 Segrate, Milan, Italy; igea.dagnano@cnr.it; 2 Department of Hematology, Transfusion Medicine and Biotechnologies, Laboratory of Stem Cells, Spirito Santo Hospital, 65125 Pescara, Italy

**Keywords:** hepatocellular carcinoma, extracellular vesicles, liquid biopsy, drug delivery

## Abstract

Hepatocellular carcinoma (HCC) is the sixth most common cancer and the third highest cause of mortality from cancer, largely because of delays in diagnosis. There is currently no effective therapy for advanced stage HCC, although sorafenib, the standard treatment for HCC, systemic therapy (including tyrosine kinase inhibitors and anti-angiogenesis agents), and more recently, immunotherapy, have demonstrated some survival benefit. The measurement and modification of extracellular vesicle (EVs) cargoes—composed of nucleic acids, including miRNAs, proteins, and lipids—holds great promise for future HCC diagnosis, prognosis, and treatment. This review will provide an overview of the most recent findings regarding EVs in HCC, and the possible future use of EVs as “liquid biopsy”-based biomarkers for early diagnosis and as a vehicle for targeted drug-delivery.

## 1. Introduction

Hepatocellular carcinoma (HCC) is one of the deadliest cancers worldwide, and accounts for 75–85% of primary liver cancer [1]. The majority of cases occur where medical and social care resources are often limited, in particular in East Asia and sub-Saharan Africa [1]. HCC constitutes a heterogeneous group of disorders that includes benign, dysplastic, and malignant lesions. In HCC management, the integration of clinical, radiological, and pathological data permits better diagnosis and the elaboration of the most appropriate treatment plan. Several studies suggested that early diagnosis significantly improves overall survival rate in HCC [1]. Although some of the biological mechanisms underlying HCC progression are well understood, and involve the tumor microenvironment, tumor-stromal interaction, epithelial–mesenchymal transition, cancer stem-cells, and the deregulation of microRNAs, many questions remain unanswered. No doubt, a better understanding of the biology of HCC will permit the development of reliable prognostic biomarkers and novel, effective therapeutic strategies [1]. In fact, traditional chemotherapy and radiotherapy only has a limited efficacy in HCC, with over 67% of HCC patients progressing to advanced stages of the disease. The measurement of α-fetoprotein (AFP) has been widely used for early detection of HCC, but increased AFP levels are also present in other diseases [2]. Since 2007, Sorafenib, a small molecule inhibitor, has become the standard treatment for HCC, while more recently tyrosine kinase inhibitors, anti-angiogenesis agents, and immunotherapy have been used [3]. From a clinical perspective, an ideal biomarker for HCC would enable diagnosis of asymptomatic HCC patients and would be used in screening asymptomatic individuals. The use of cancer genomics for diagnosis has proven to be of enormous utility in cancer medicine, due to the rapid development of personalized clinical approaches based on targeted therapy directed against specific molecular alterations. The potential of a personalized approach promises to increase the efficacy of treatment with minimal toxicity and cost.

During the past few years, the new concept of “liquid biopsy” has emerged and has opened up previously unexpected perspectives. A liquid biopsy is a revolutionary, minimally-invasive technique performed on samples of blood or other human fluids to detect and quantify circulating tumor cells (CTCs) or other tumor elements, such as cell-free tumor DNA (ctDNA) and extracellular vesicles (EVs), which are released in the blood or other human fluids by cancer cells. Thus, these tumor elements can be detected in the body fluids as a source of tumor tissue information in patients with different tumor types, including HCC [4,5]. Evaluation of CTCs, ctDNA, cell-free tumor DNA methylation, and circulating RNA as possible liquid-biopsy-based biomarkers in HCC could help improve the early diagnosis of HCC, in particular when it is multifocal or advanced, which are the most heterogeneous HCCs [6].

In the last decade, nanoparticles known as extracellular vesicles (EVs) have been included as possible components of liquid biopsy tests in many human diseases. Since their discovery in the 1970s, researchers have only recently focused their attention on EVs [7]. Increasing evidence has demonstrated the presence of EVs in all main body fluids, such as blood, urine, bile, and saliva [8,9]. Their presence in biological fluids and their distinctive composition in proteins, RNA, and lipids render EVs biomarker candidates of great significance in cancer, including HCC [10,11,12]. This review provides a summary of the recent reports on the role of EVs in HCC progression and the expanding role of EVs in systemic treatment of HCC, highlighting emerging strategies that promise future therapeutic options.

## 2. Overview of Extracellular Vesicles

It is now widely accepted that intercellular communication occurs not only through direct contact between cells, for example mediated by tight junctions or proteins [11,13,14] or through soluble factors (proteins or lipids) in an autocrine, paracrine, and endocrine scenario; but also through released EVs [15].

EVs are lipid bilayer-delimited nanoparticles secreted by the cells by different complex mechanisms. They derive from diverse kinds of membranes at two different subcellular sites—the plasma membrane and the endocytic membranes [16,17]. They are highly heterogeneous, and can be mainly classified according to MISEV (Minimal Information for Studies of Extracellular Vesicles) 2018 guidelines into small EVs (also called exosomes, with sizes <100 nm or <200 nm) and large EVs (also called microvesicles (MVs) or ectosomes, with sizes >200 nm) [18,19].

The different ways of producing EVs suggest that the various EV populations are different from each other. Different attempts have been made to isolate pure populations of large and small main types of EVs.

Exosomes are those EVs produced inside cells by the internal budding of the membrane of endocytic cisternae during early endosome formation, and their precursors are called intraluminal vesicles (ILVs). When ILVs accumulate, they form multivesicular bodies (MVBs) that are then secreted, fusing with the cell membrane [18,20,21]. Conversely, MVs arise from the extroversion of the plasma membrane, causing MV blebbing and release in the extracellular area [22].

However, there are exosomes that are larger than 200 nm and MVs that are smaller than 200 nm. At present, no unique markers have been identified to differentiate exosomes from other nanoscale vesicles, and this could evidently limit their specific detection in human fluids [11].

EV cargo is constituted of proteins (both soluble and as a part of the EV membrane), RNA, lipids, and metabolites that can be conveyed by the EVs themselves to nearby or distant cells [18]. Cell conditions and external stimuli can influence the specific EV cargo composition, suggesting that the cells are able to select the EV packaging, safeguarding the molecules packaged into the EVs from enzymatic degradation. For example, serum starvation can modify EV cargoes [23]. Liem et al. demonstrated that insulin activates a signaling pathway modifying the EV protein cargo [24]. It has been also reported that upon stress stimuli tumor cells may influence the cargo of secreted EV as a survival strategy [25]. Hypoxia may induce peculiar secretory EV phenotypes by increasing the release of EVs [25,26]. Moreover, the intracellular pH can affect the biogenesis of EVs, and in particular an acidic pH can increase their secretion [25,27]. It has also been reported that endoplasmic reticulum stress is associated with secretion of EVs with different molecular compositions [28,29]. Also, it is known that calcium signaling is crucial for different stages of tumor progression, partly due to its effects on the biogenesis of EVs [25,30].

Indeed, EVs are able to spread oncogenic messages through RNAs and proteins that regulate important processes inside the cells, such as proliferation, differentiation, survival, and migratory ability in different tumor types, including HCC [31,32,33]. Finally, EVs also participate in inflammatory processes, angiogenesis, extracellular matrix remodeling, the formation of the metastatic niche, and inhibition of the antitumor immune response [11].

## 3. Extracellular Vesicles in Hepatocellular Carcinoma

The liver is composed of several different cell types, including cancer-associated fibroblasts (CAFs), Kupffer cells, hepatic stellate cells (HSCs), endothelial cells, infiltrating immune cells, recruited mesenchymal stem cells (MSCs), and cancer cells, each capable of producing different types of EVs, contributing to interactions between them. Since different stages of liver disease are associated with different EV cargoes, EV profiling has potential diagnostic and prognostic value in HCC [11,34]. Increasing number of reports indicate that EVs also have an important role in liver metastasis from other tumor types, preparing the premetastatic niche to receive metastatic tumor cells, creating a favorable environment for the propagation of metastatic tumor cells [35,36,37]. EVs derived from HCC cells contribute in cell-to-cell communication and have been demonstrated to regulate various cellular processes [38,39]. There is some evidence indicating that EVs isolated from HCC patients are able to mediate resistance to sorafenib [40,41]. Gene profiling and protein expression analysis of EVs isolated from metastatic HCC cells identified the mesenchymal-epithelial transition (MET) proto-oncogene S100 family members and caveolins as tumor-promoting molecules in HCC [42,43]. Considerable evidence has indicated that miRNAs contained in EVs contribute to metastasis, chemoresistance, and immunomodulation in different cancer types, including HCC [44]. Min Shi and co-authors have studied the diagnostic and prognostic use of serum EV-miR-638 in HCC patients. They found lower levels of this miRNA in the serum of HCC patients compared to healthy donors. Moreover, HCC patients with reduced serum levels of EV-miR-638 had poor survival at both 3 and 5 years [45]. The downregulation of tumor suppressor miRNAs such as miR-638 is a mechanism used by many tumor cells to coordinate activation of the metastatic cascade, thus increasing their survival advantage during tumor progression. Thus, its downregulation in serum EV represents a valuable marker in HCC.

Fang et al. found that the release of EV-miR-1247-3p in the circulation is related to the level of metastatic potential in HCC cells. The authors also demonstrated that serum levels of EV-miR-1247-3p correlate with the presence of lung metastasis in HCC patients, and this is due to the ability of this miRNA to trigger the β1-integrin–NF-κB signaling pathway in a crosstalk between cancer cells and fibroblasts, transforming fibroblasts into CAFs in the lung premetastatic niche [46]. These authors further clarified the molecular mechanism of lung metastasis from liver cancer, and explained why diverse liver cancer types show different abilities to induce lung metastasis.

Li and colleagues demonstrated a novel function of a member of the lysyl oxidase (LOX) family of proteins, LOXL4, in HCC exosome-mediated tumor metastasis, identifying a new promising therapeutic target for HCC. They found that exosomal LOXL4, by triggering the FAK/Src pathway, has oncogenic properties in HCC through promotion of migration, invasion, and metastasis, and predicts a poor prognosis. [47].

Tang and colleagues evaluated the possible diagnostic value of exosomal miR-9-3p in HCC. They detected this miRNA in the blood of HCC patients with a high sensitivity and specificity, showing lower levels of serum exosomal miR-9-3p in HCC patients than in healthy donors. This could help identify miR-9-3p as a potential therapeutic target for HCC [48].

Next-generation sequencing was used to discriminate liver cirrhosis from HCC. The authors found that miR-122, miR-148a, and miR-1246 were significantly elevated in serum EVs from HCC patients compared to liver cirrhosis patients, who are at high risk for HCC, as well as compared to normal individuals [49]. These findings could be helpful for prediction of liver disease progression and for assessing possible preventive treatments of HCC.

It has been demonstrated that miR-93 promotes cell proliferation by regulating the Phosphatase and TENsin homolog (PTEN) in several types of cancer [50,51]. Xue et al. showed that serum EV-miR-93 was also significantly increased in HCC patients and correlates with clinical features, including tumor stage, size, and survival rate, suggesting that this miRNA could be employed as a novel diagnostic and prognostic biomarker in HCC [52].

In addition, EVs in the blood contain a considerable number of valuable and functional extracellular vesicles Long RNAs (exLRs). Li et al. showed the potential of exLR-based liquid biopsy for diagnosis of different types of cancers. In particular, they demonstrated that exLRs could serve as highly sensitive and specific diagnostic biomarkers in HCC. They were able to diagnose early-stage or alpha-fetoprotein (AFP)-negative HCC and distinguish HCC patients from those with hepatitis, liver cirrhosis, or benign tumors. In their paper, the authors also established a large-scale blood exLR atlas of healthy individuals and cancer patients, which could represent a very useful tool for screening studies [53].

Another class of interesting noncoding RNAs enriched in EVs is represented by circular RNA, which are more abundant than their canonical linear transcripts because of their high stability. The circRNA are released by cells enriched in EVs and have been detected in many body fluids. In HCC, many circRNAs are deregulated, and their altered expression is often correlated with the clinical pathological characteristics. The paper by Wang et al. found three isoforms of circPTGR1 enriched in EVs isolated by a highly metastatic HCC cell-line, which were also found to be related with the clinical stage and prognosis in HCC patients. Further, circPTGR1 may influence recipient cells with lower malignancy by affecting miR-449a–MET interactions. Thus, by disrupting the homeostasis of the microenvironment, circPTGR1 may promote HCC progression. For these reasons, circPTGR1 could represent a prognostic biomarker that should be considered as a therapeutic target in HCC [54].

Interestingly, Ye and colleagues demonstrated that EVs isolated from HCC cells can stimulate the accumulation of TIM-1 + Breg cells, recruiting the HMGB1-TLR2/4-MAPK pathway. TIM-1 + Breg cells create a microenvironment in which IL-10 is secreted and the activity of CD8+ T is impaired, thus favoring HCC progression. These data could be the basis for the development of immunotherapy strategies in HCC [55].

In Table 1, the main references reported in this section are summarized, specifying EV isolation techniques, biomarkers, and functions.

Due to their central role in cell-to-cell communication, EVs can be targets for therapy at different levels of their biogenesis and uptake by recipient cells. For example, blocking or removing cancer-derived EVs could constitute a potential therapeutic approach. It is noteworthy that EVs could also be used as carriers for proteins, RNAs, metabolites, and drugs with antitumor activity.

## 4. EVs as a Basis for a Therapeutic Approach

Fundamental questions remain unsolved regarding the mechanisms regulating EVs in the liver. To achieve a deeper understanding of the production of EVs, the EV target cell repertoire, EV receptors, and the range of action of EVs are under investigation. Several studies are also exploring the possible utilization of EVs in the treatment of liver cancer. Differing approaches toward how therapeutic molecules can be delivered effectively using EVs are currently under investigation, including the use of EVs of different origins. A brief summary of recent, promising developments potentially useful for treating HCC will be given here. In order to enhance drug efficacy and limit side effects, EVs have recently been studied as vehicles to deliver targeted drugs. A variety of therapeutic agents can be loaded onto EVs, which include chemotherapeutic agents and nucleic acids [56]. In particular, the challenging delivery to target cells of nucleic acids, due to their low intracellular uptake and susceptibility to enzymatic degradation in extracellular space, makes EVs an excellent promising carrier for these molecules [56]. Indeed, recent studies have shown effective delivery of EV-packaged drugs to target tumor sites with better pharmacokinetic efficiency and therapeutic efficacy.

Furthermore, many research studies have shown that similar to in other types of cancer, intercellular EV-mediated communication between cancer and stromal cells play an important role in liver cancer progression through cargo transfer. Consequently, cancer derived-EVs, including HCC, are the focus of several investigations attempting to reduce EV cargo transmission through the inhibition of EV production, the elimination of circulating EVs, and the inhibition of EV uptake [57].

It should be noted that drawing wider conclusions based on EV drug loading investigations is complex, as the results obtained will be depend on the source of the EVs used, the isolation technique, the choice of therapeutic agent, and the loading protocols used. Furthermore, EVs harvested from biological sources are highly heterogeneous, making it difficult at this time to precisely control EV purity and quality as carriers for treatment.

### 4.1. Loading of Therapeutic Agents to EVs

Among the various technologies utilized, “preloading techniques” foresee the incorporation of a desired cargo in cells, which will subsequently “encapsulate” this material during the production of the EVs. Cells are able to package not only components that are produced biologically, such as nucleic acids and proteins, but also synthetic compounds. The treatment of cancer cells using chemotherapeutic agents, for example, will lead to production of EVs, which carry drugs. Tang et al. preloaded doxorubicin in an HCC cell-line, obtaining EVs packaging the chemotherapeutic agent. Subsequently, the HCC cell-line-derived EVs were used as a carrier to deliver doxorubicin to tumor cells, demonstrating that the doxorubicin carried by EVs is more effective than dispersed doxorubicin in inhibiting tumor growth and prolonging survival time in HCC xenograft mice models, with reduced side effects [58]. Very recently, in order to enhance iodine (I) avidity in HCC cells, Son and colleagues investigated a novel approach for transferring the sodium/iodide symporter (NIS) protein to cells using EVs. HCC cells were transfected (Huh7 cell line) with NIS gene and EVs were isolated from them. Results demonstrated that the NIS protein had been successfully transferred into Huh7 cells with isolated EVs displaying high NIS protein levels. Furthermore, treating HCC cell lines with Huh7-derived EV–NIS increased levels of the NIS protein and enhanced the uptake of ^125^I in the HCC recipient cells. Additionally, the pretreatment of Huh7-derived EV–NIS increased ^131^I therapy’s cytotoxicity against the HCC cell line by causing an increase in damage to DNA and an increase in the formation of γH2A.X foci. Using this innovative approach, cancer can revert from being resistant to radioiodine to being sensitive to it. These results suggest a potential use of EVs as platforms for NIS transfer in radioiodine-based therapies on cancers which are currently resistant to radioiodine, or cancer cells that do not express NIS or which have reduced expression of NIS, thus improving anti-cancer treatment [59].

“Mixing with free drugs” is one reliable strategy, which avoids the use of more complex solutions for incorporating therapeutic agents in EVs. Methods for active loading of EVs can be separated in two categories: those that can be induced physically, and those that can be induced chemically. Drug loading induced physically is performed by mechanically disrupting EV membranes. Drug loading induced chemically makes use of chemical agents to bypass the EV membrane. Pomatto et al. used plasma-derived EVs to promote apoptosis (using HepG2). First, miR-31 was loaded into EVs derived from plasma using electroporation. Consequently, EVs incorporating miR-31 by electroporation were able to silence CDK2, which is overexpressed in HCC and appears to play a role in the mechanisms regulating cell cycle and SP1. Furthermore, the SP1 gene itself has an important role in the mechanisms regulating cell apoptosis, cell proliferation, and cell invasion in HCC [54]. Similarly, miR-451a, which targets *BCL2α,* when loaded into EVs by electroporation also reduces the expression of *CASP3*, and thus has a role in inducing the apoptosis of HepG2. EVs that have miR-451a incorporated are able to suppress expression of one of its target genes, *MDR1.* It is known that *MDR1* regulates chemosensitivity, not only in HCC but also in a number of other types of cancer [60].

### 4.2. Different Sources of EVs for Therapeutics

Wang et al. reported that miR-335-5p was incorporated into EVs using Lipofectamine RNAiMAX reagent (ThermoFisher Scientific, Waltham, MA, USA). These EVs carrying miR-335-5p were used against tumor cells in both in vitro and in vivo models. Interestingly, they incorporated miR-335-5p into EVs derived from stellate cells, showing the uptake of these EVs on HCC cells and demonstrating the transfer of the miR-335-5p cargo to the HCC cells. The EVs carrying miR-335-5p inhibited the proliferation and the invasion of HCC cells. Similarly, they were also able to reduce HCC tumor size in vivo [61]. An interesting aspect of this study is the demonstration that EVs could be produced by other types of cells aside from the cancer-originating cell type, and that EVs produced from stellate cells had certain characteristics that were related to the originating cell. This is of great significance for developing cancer therapies using EVs in the future. Another interesting approach was developed by Liang et al., who engineered human cell-line-293T-derived EVs to deliver specific therapeutic miR-26a to an HCC cell line, demonstrating the capacity of these engineered EVs to be able to suppress the HepG2 cell in vitro. Furthermore, they modified the EV with Apo.A1 (apolipoprotein A1) to specifically target the HepG2 cell line through the receptor SR-B1, which is abundant on the surface of HCC cells. They demonstrated that this modification increases the efficiency of the uptake on the target HCC cell line and the specificity and efficiency of the release of miR-26 into the cytoplasm of the HCC cell lines [62].

The use of dendritic cell (DC)-derived EVs in clinical trials has been shown to induce antitumor immunity. Furthermore, it would seem that tumors that are not responsive to immunotherapy could be successfully treated using DC-derived EVs. Antigen-expressing DC-cell-derived EVs were demonstrated to have an antitumor function in three pathologically and antigenically heterogeneous HCC mouse models, inducing an antigen-specific powerful immune response, leading to longer survival. It has been proven in an in vivo model that DC-derived EVs can induce and improve T-cell responses that are antigen-specific, and clinical trials have already demonstrated that the use of EVs derived from DCs is both feasible and safe for a variety of types of tumor. Furthermore, it has recently been shown that DC-derived EVs have an antitumor immune response against HCC by inducing the proliferation of naive T-cells and activating T-cells. In addition, DC-derived, EV-sensitized precursors have been shown to be more effective at maximizing the activation of specific immune responses against HCC, inducing a restricted cytotoxic T-cell response (class I MHC complex). DC-EVs (derived from human peripheral blood) were loaded with recombinant adeno-associated viral vector (rAAV)-carrying alpha-fetoprotein (AFP) genes (*rAAV*/*AFP*). A diethylnitrosamine-induced HCC model gave the most significant antigen-specific, antitumor immune response from EVs derived from DCs expressing AFP. By inducing activation of CD8^+^ cytotoxic T-lymphocytes and the increased expression of IFN-*γ* and IL-2, reducing the expression of regulatory T-cell CD25^+^/Foxp3 and decreasing the expression of IL-10 and TGF-*β*, EVs derived from DCs expressing AFP are more effective at tumor immune-suppression, thus performing the role of a “cell-free vaccine” [63]. Thus, DC-derived EVs offer an alternative route for HCC immunotherapy through tumor-specific antigen modification.

A small subset of cancer stem cells (CSCs) are present in the HCC microenvironment, which have been shown to have a significant role in the development and progression of HCC [64]. CSCs probably derive from a malign transformation of normal stem cells that reside in the liver [64]. Importantly, CSC-derived EVs can mediate chemoresistance and tumor metastasis. These CSC-derived EVs should be targeted to improve tumor eradication. One very recent study investigated whether the transfer of EVs deriving from hepatic CSC and bone-marrow-derived mesenchymal stem cell (MSC) EVs induced or inhibited tumor growth and metastasis in the HCC microenvironment, according to their source of origin. CSC-derived EVs caused a noteworthy increase in the relative weight of the liver and in the levels of AFP and gamma glutamil transferasi (GGT) cancer markers in the serum, as well as increased levels of amino alanine transferase (ALT), aspartate aminotransferase (AST), and alkaline phosphatase (ALP) liver enzymes. Further, increased expression of the glutathione S-transferase (GST)-P marker for HCC was found, as were a greater number of tumor nodules covering a larger area. These results were obtained in rats bearing HCC compared with control rats injected with phosphate-buffered solution (PBS) [65]. Additionally, CSC-EVs reduced apoptosis, increased angiogenetic activity, enhanced metastasis and invasiveness, and led to epithelial mesenchymal transition, increasing the expression of TGF*β*1 in both the serum and a hepatic sample. Notably, CSC-EVs also raised levels of HCC-EV miR-21, EV lncRNA Tuc339, long noncoding High Expression In HCC (lncHEIH), and the HCC long noncoding Hox transcript antisense intergenic RNA (lncHOTAIR), and lowered liver miR-122 and HCC miR-148a, miR-16, and miR-125b. All the effects mentioned above were reversed by the injection of bone marrow (BM) MSC EVs. Collectively, these results revealed that CSC-derived EVs induced HCC tumor development and progression, whereas MSC-derived EVs had an inhibitory effect [65]. MSC-secreted EVs represent a great opportunity for research into therapeutic applications and effectiveness in combating tumors, thanks to their antitumor activity and their immunomodulation effect [66]. A number of studies have suggested that administration of EVs derived from MSCs can ameliorate the negative side effects of acute liver injury and liver fibrosis [67]

As mentioned above, one important question for cancer treatment regards chemotherapeutic drug resistance. In a variety of HCC cell populations, EVs have been proven to mediate horizontal transfer of chemoresistance. Sorafenib has been used in clinical HCC therapy and offers a better prognosis for patients with HCC. In cancer cells that are resistant to sorafenib, GRP78 is overexpressed as compared to cells that are sensitive to it, suggesting that it could be a target for HCC treatment. In a recent study, BM MSCs were modified to produce EVs expressing siGRP78, which plays crucial roles in sorafenib resistance. EVs expressing siGRP78 used in combination with sorafenib effectively targeted GRP78 in HCC cells, as well as inhibited tumor growth and invasion, both in vitro and in vivo. Therefore, EVs modified with siGRP78 were able to reverse sorafenib drug resistance [41].

### 4.3. Cancer Derived-EV-Release Inhibition

As mentioned before, the development of inhibitory techniques limiting cancer-derived EV release is an important area of research. Recently, Kosgodage et al. [68] reported that cannabidiol (CBD) plays a role in inhibiting the release of EVs from an HCC cell line (HepG2). CBD, which is a phytocannabinoid extracted from *Cannabis sativa*, possesses antioxidant, anti-inflammatory, and antiproliferative properties. Moreover, their data suggest that CBD, by modulating the expression of the mitochondria-associated proteins, STAT3, and prohibitin, is also able to affect mitochondrial function.

**Figure 1:**
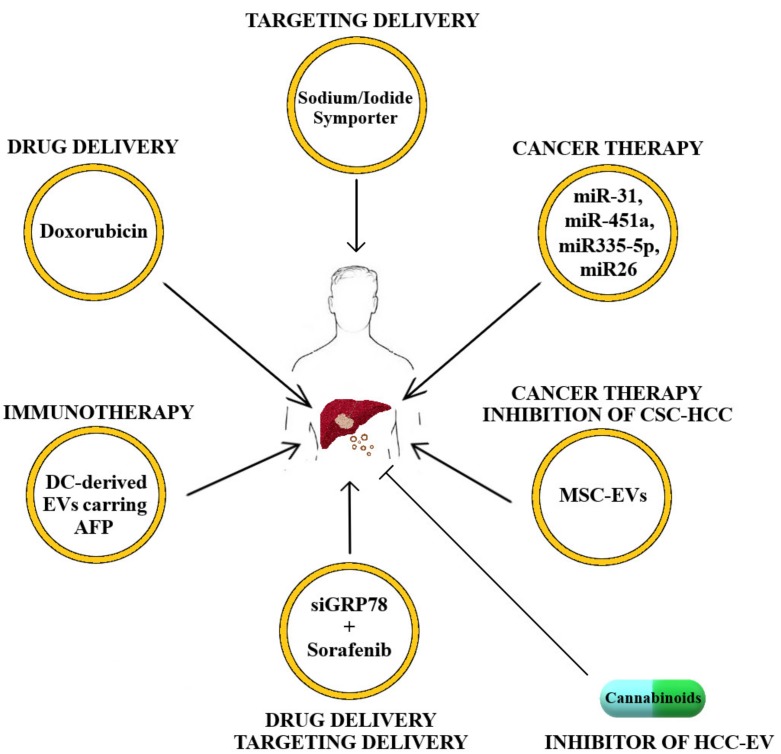
Schematic representation of extracellular vesicle (EV) therapeutic strategies for hepatocellular carcinoma (HCC) treatment. Note: CSC = cancer stem cell; MSC EVs = mesenchymal stem cell (secreted EVs); DC = dendritic cell; AFP = alpha-fetoprotein).

The main therapeutic strategies for HCC reported in this review are graphically summarized in Figure 1.

## 5. Conclusions

**Figure 2:**
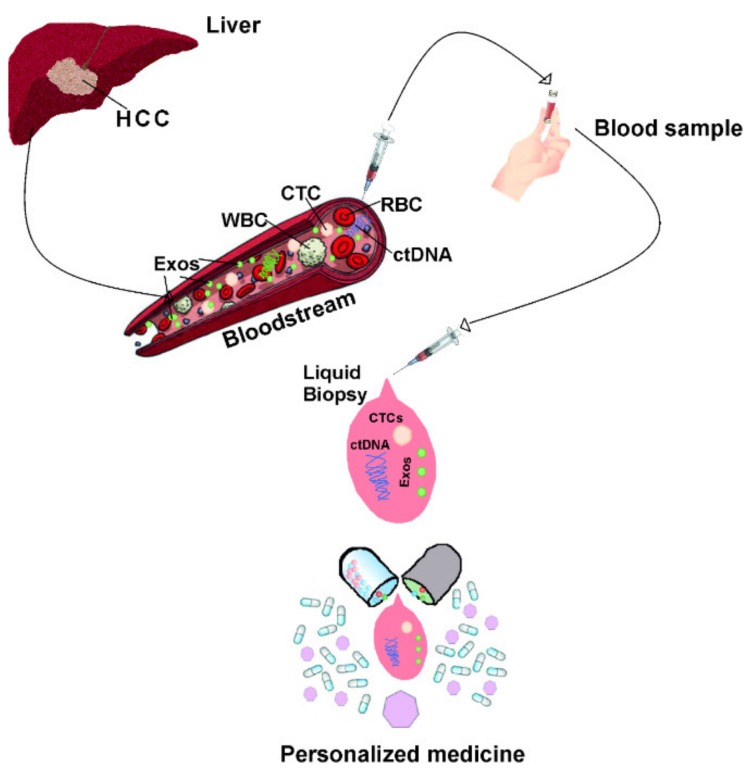
Graphical scheme of the possible therapeutic platform that translates EVs for liquid biopsy and drug delivery in the scenario of personalized medicine. Note: HCC = hepatocellular carcinoma; CTC = circulating tumor cells; RBC = red blood cells; WBC = white blood cells; Exos = exosomes, ctDNA = circulating free tumor DNA.

The translation of EVs into a therapeutic platform for liquid biopsy and drug delivery may be realized in the very near future (Figure 2). Therefore, it is crucial to clarify the mechanisms underlying HCC’s distinct pattern of progression and metastasis. Emerging evidence has shown that EVs mediating the intercellular communication that occurs between HCC cells and the surrounding microenvironment facilitate tumor cell dissemination. Furthermore, studies have recently suggested the great potential of both EV-targeting and EV-based therapies.

In this review, we have sought to describe the wide variety of different roles that EVs can play in HCC pathogenesis and progression, along with their possible use in potential treatments. It should, however, be emphasized that in addition to the many unsolved questions that remain to be answered, some of which are mentioned above, methods for the isolation and characterization of EVs still need to be standardized. This is not only important for research itself, but is also fundamental in order to permit use of EVs in clinical practice. Standardized methodologies are required for the storage of EVs, for clinical diagnosis, and for the realization of personalized HCC treatments, together with all appropriate quality control protocols for clinical EV production. A guideline entitled “Minimal Information for Studies of Extracellular Vesicles, 2018 (MISEV2018)”, suggesting minimum standardized methodological requirements, has recently been published by the International Society for Extracellular Vesicles [19]. There is great heterogeneity among EVs and a large number of in-depth studies will be needed in order to fully understand their biogenesis, uptake, secretion, and even their cargo composition. Genomic and associated proteomic studies are required together with functional studies to reach a deeper understanding for the development of personalized medicine at both diagnostic and therapeutic levels, not only in HCC, but also in other forms of cancer in order to optimize clinical translation. All of these studies will permit possible identification of one or more specific EV subpopulations for use in targeted drug delivery according to physical-chemical properties, loaded drug capacity, and specific degree of uptake by target cells. It is important to highlight that an optimal technique for incorporating small molecules, pharmacological drugs, or nucleic acids into the vesicles has not yet been identified. It is also important to underline that the examples of methods of incorporation mentioned in the previous paragraphs are highly dependent on the type of cargo to be incorporated and on the source of the EVs that are used. In fact, all these variables lead to conflicting results and low incorporation efficiency. All of these considerations, taken together, lead us to conclude that it will likely be necessary to select the most appropriate approach or combination of approaches according to the particular advantages of each loading technique. As far as the utilization of EVs for drug delivery is concerned, it is necessary to confirm which EV source is the most appropriate. Optimizing EV use efficiency and avoiding any immunotoxic reaction would also require full understanding of any variation between the use of autologous and allogenic EVs. A multidisciplinary approach is of fundamental importance, including the development of engineering technologies, the importance of which is already being demonstrated using synthetic materials with EVs. Rigorous investigation under clinical conditions is fundamental, not only to delineate the full therapeutic potential of EVs, but also to confirm safe use, clarifying the mechanisms and underlying pathways that are pivotal for EV-mediated DNA, mRNA, miRNA, lncRNA, and siRNA drug delivery and processing. Further investigation will clarify many unsolved questions about the role of EVs in HCC development and progression, and future clinical applications.

## Figures and Tables

**Table 1:** Summary of most recent isolation techniques, biomarkers, and functions of extracellular vesicles (EVs) in hepatocellular carcinoma (HCC).

Ref.	EV ISOLATION METHOD	BIOMARKER	FUNCTION
46	Differential ultracentrifugation	miR-1247-3p (target, B4GALT3)	Activation of β1-integrin–NF-κB signaling in fibroblasts; correlation with lung metastasis in HCC patients
52	Total exosome isolation kit (Thermo Fisher Scientific).	miR-93 (targets,CDKN1A,TP53INP1, and TIMP2)	Promotes HCC tumorigenesis, predicts poor prognosis
49	8% Polyethylene glycol (PEG) 6000 (Sigma-Aldrich, St Louis, MO, USA)	miR-122, miR-148a, and miR-1246	Combination of exosomal microRNAs and alpha-fetoprotein (AFP), yielded a better diagnostic power than AFP in discriminating subjects with early HCC from liver cirrhosis
39	ExoQuick-TC kit	NKG2D, HSP70	Play important roles in angiogenesis
48	Differential ultracentrifugation	miR-9-3p (target, HBGF-5)	Downregulated HBGF-5 expression at both the mRNA and protein levels
45	Total exosome isolation kit (Invitrogen, Carlsbad, CA, USA).	miR-638	The downregulation predicts poor prognosis for HCC patients
40	Differential ultracentrifugation	miR-325p	Activates the PI3K/Akt pathway and induces multidrug resistance by modulating angiogenesis and epithelial-mesenchymal transition (EMT)
42	Life Technology exosome precipitation solution (Invitrogen, Carlsbad, CA, USA)	miR-320a (target, PBX3)	Low expression drives the cancer cells towards a more malignant phenotype
47	Differential ultracentrifugation	LOXL4	Enhances the invasive potential of HCC cells, promotes angiogenesis, thus facilitating HCC metastasis
53	N.A.	lncRNA, circRNA	May diagnose early-stage or AFP-negative HCC; distinguishes HCC patients from those with hepatitis, liver cirrhosis, or benign tumors.
38	Exoquick Exosome Precipitation Solution (System Biosciences)	miR-155	Up-regulation induces tube formation of human umbilical vein endothelial cells (HUVECs) and affects angiogenic activity and recurrence in hepatocellular carcinoma
54	ExoQuick-TC exosome precipitation solution (System Biosciences, CA, USA)	circPTGR1	Increases the migratory and invasive abilities

N.A. = not available.
